# CRISPR/Cas9-mediated genome editing induces exon skipping by complete or stochastic altering splicing in the migratory locust

**DOI:** 10.1186/s12896-018-0465-7

**Published:** 2018-09-25

**Authors:** Dafeng Chen, Ji-Xin Tang, Beibei Li, Li Hou, Xianhui Wang, Le Kang

**Affiliations:** 1 State Key Laboratory of Integrated Management of Pest Insects and Rodents, Institute of Zoology, Chinese Academy of Sciences, Beijing, 100101 China; 2 Affiliated Hospital of Guangdong Medical University, Zhanjiang, 524001 China; 3 University of Chinese Academy of Sciences, Beijing, 100049 China

**Keywords:** CRISPR/Cas9, Gene editing, Exon skipping, Migratory locust, Pre-mRNA splicing

## Abstract

**Background:**

The CRISPR/Cas9 system has been widely used to generate gene knockout/knockin models by inducing frameshift mutants in cell lines and organisms. Several recent studies have reported that such mutants can lead to in-frame exon skipping in cell lines. However, there was little research about post-transcriptional effect of CRISPR-mediated gene editing in vivo.

**Results:**

We showed that frameshift indels also induced complete or stochastic exon skipping by deleting different regions to influence pre-mRNA splicing in vivo. In the migratory locust, the missing 55 bp at the boundary of intron 3 and exon 4 of an olfactory receptor gene, *LmigOr35*, resulted in complete exon 4 skipping, whereas the lacking 22 bp in exon 4 of *LmigOr35* only resulted in stochastic exon 4 skipping. A single sgRNA induced small insertions or deletions at the boundary of intron and exon to disrupt the 3′ splicing site causing completely exon skipping, or alternatively induce small insertions or deletions in the exon to stochastic alter splicing causing the stochastic exon skipping.

**Conclusions:**

These results indicated that complete or stochastic exon skipping could result from the CRISPR-mediated genome editing by deleting different regions of the gene. Although exon skipping caused by CRISPR-mediated editing was an unexpected outcome, this finding could be developed as a technology to investigate pre-mRNA splicing or to cure several human diseases caused by splicing mutations.

**Electronic supplementary material:**

The online version of this article (10.1186/s12896-018-0465-7) contains supplementary material, which is available to authorized users.

## Background

Clustered Regularly Interspaced Short Palindromic Repeats/CRISPR associated protein 9 (CRISPR/Cas9), which is a rapid and efficient system to generate genome mutation, has been used in many organisms for gene function loss [1–9]. Short frameshift insertion-deletions (indels) are usually introduced in exonic sequences to disrupt the reading frame of mRNA by CRISPR/Cas9. These indels are created by an endogenous DNA repair machinery via non-homologous end joining (NHEJ) when the Cas9 nuclease generates double-strand breaks (DSB). Currently, many studies have focused on frameshift mutation by generating DSB for genome editing purposes. One major application of CRISPR/Cas9 system is to generate inactivating mutations in protein-coding genes by targeting single sgRNA sites to create frameshifts. Most of the indels in protein-coding gene exons are supposed to be frameshift mutations disrupting open reading frames with the obvious exception of those whose size is multiple of three. Frameshift indels are very suitable for generating loss-of-function mutations in protein coding genes. These mutated transcripts are recognized and degraded by a nonsense-mediated mRNA decay (NMD) machinery or are translated into truncated non-functional proteins.

Pre-mRNA splicing is catalyzed by the spliceosome, one of the largest ribonucleoproteic complex of the cell. Through splicing intronic sequences of pre-mRNA are eliminated from pre-mRNA and exonnic sequences are joined together. Pre-mRNA splicing requires several *cis*-acting elements on the pre-mRNA: (1) 5′ donor and 3′ acceptor splice site consensus sequences, by which the exon–intron boundaries are constituted; (2) a branch point, which is consisted by an adenosine, located in a consensus sequence of the intron, 18–40 nucleotides upstream of the 3′ acceptor splice site [10]. The 5′ and 3′ splice site and branch point is essential for the pre-mRNA splice, therefore they should be considered when we generated the frame-shift mutant by CRISPR/Cas9 system.

Recently, several studies have reported the unintended consequences at the post-transcriptional level, such as aberrant RNA splicing, caused by CRISPR-mediated editing of the target gene [11]. The insertion of a large DNA fragment into an exon of human *hCDC14A* and *hCDC14B* genes by genome editing introduced its skipping from the final transcript in human hTERT-RPE1 and HCT116 cells [12]. A single base change in the target exon of human FLOT-1 gene resulted in random splicing in HeLa cells [13]. The frameshift indels engineered by CRISPR/Cas9 also led to skipping of “multiple three nucleotides” [14]. A single sgRNA induced partial exon splicing or unexpected large deletions that removed exons [15]. These in vitro studies on cell lines revealed other artifactual effects of CRISPR applications aside from their off-target effects, thereby providing new information for better mutant allele screening. Recent study in zebrafish showed that disrupted ESE by inserted 7 bp nucleotides could resulted into exon skipping [16]. These results suggest that stochastic exon skipped induced by the indels in exon can be found both in vivo and in vitro. However, few of in vivo studies have been designed specifically to disrupt the *cis*-acting elements (such as the 5′ and 3′ splice sites) by CRISPR-mediated genome editing due to the complexity of the *cis*-acting elements during the pre-mRNA splicing in organisms [17]. Besides the 5′ and 3′ splice sites there are many *cis*-acting elements (such as splicing enhancers or silencers in exons or in introns) that can influence the pre-mRNA splicing. Therefore, it is difficult to identify the *cis*-acting elements and to disrupt them precisely by genome editing.

The CRISPR/Cas9 system has been successfully applied to generate knockout mutant lines in the migratory locust, *Locusta migratoria* [7], which has served as model species for phenotypical plasticity involved in behavior, morphology, and physiology [18–24]. The genome sequencing of locust showed that this insect had a huge genome (6.5 Gb) and displayed the unique characteristics on the splicing mechanisms of long introns compared with other insect species [25]. The unique characteristics of locust genome were mainly in proliferation of a diverse range of repetitive elements, the lowest divergent of DNA transposon and big intron and so on [25]. Previous reports have indicated that most insects have an enrichment of ratcheting point sites to allow for efficient splicing of long introns, whereas vertebrates use repetitive elements to aid in splicing long introns and the splicing mechanisms may be convergent evolution associated with the genome size expansion in animals [26]. Probably, locusts is a potential model for studying the effect of gene editing on exon splicing in large genome organisms by CRISPR/Cas9 system.

An olfactory receptor gene suitable for investigating gene editing was selected to perform splicing disruptions in vivo, because the knockout of these olfactory receptors (Ors) was not lethal. Here, we showed that CRISPR-mediated editing of one olfactory receptor gene of locusts induced complete or stochastic exon skipping. Complete exon skipping was caused by the boundary deletion of intron and exon (3′ splice site) that completely altered the pre-mRNA splicing, whereas stochastic exon skipping was due to the alternative splicing that caused by the indels in exon, which changed the cis-effector that promotes the pre-mRNA splicing.

## Results

### Deletion of one 3′ splice site of *LmigOr35* using CRISPR/Cas9 system

**Fig. 1:**
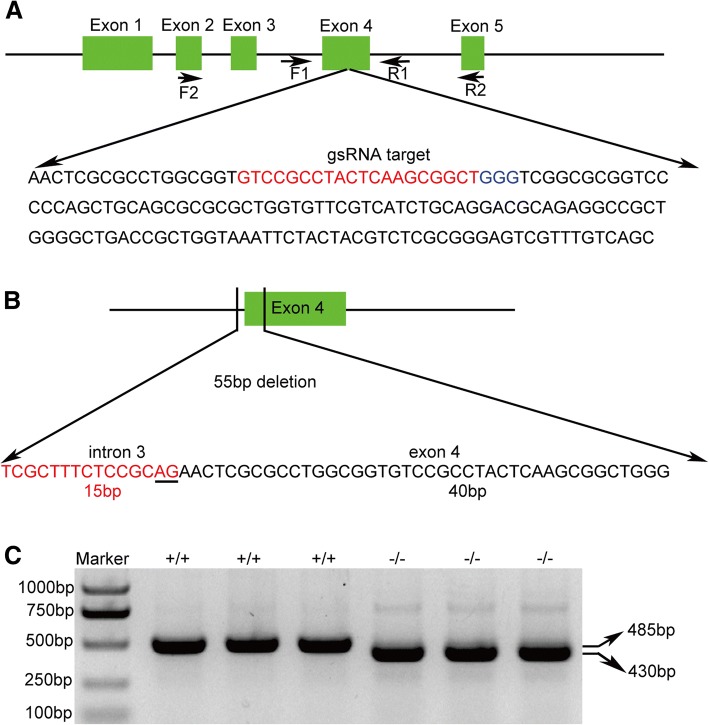
Disrupting the 3′ splice site by deletion of 55 bp nucleotides at the boundary of intron 3 and exon 4 of locust *LmigOr35* using CRISPR/Cas9 system. **a** The entire gene structure of *LmigOr35* with all introns and exons and the designed sgRNA targeted site in exon 4 of locust *LmigOr35*. F1 and R1 are primers for detecting genome deletion; F2 and R2 are primers for detecting exon deletion. **b** Deleted nucleotides containing 15 bp intron 3 and 40 bp exon 4 nucleotides of locust LmigOr35. Under line shows the conserved nucleotides in the splice site that are crucial for normal splicing. **c** Genotype of WT (+/+) and 55 bp mutant (−/−) locusts. The WT locusts obtain a 485 bp brand and the 55 bp mutants obtain a 430 bp brand

**Fig. 2:**
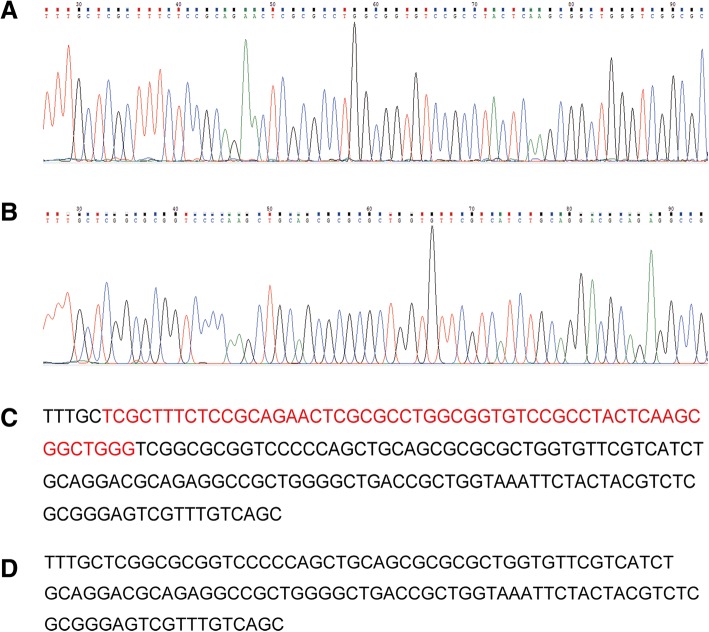
Genome PCR products sequencing of the wildtype and the 55 bp mutant locust LmigOr35. **a** and **c** is the wildtype partial locust *LmigOr35* gene sequencing trace and nucleotides sequence. Nucleotides marked with red only exist in the wild type locusts but not in the 55 bp mutant locusts. **b** and **d** is the 55 bp mutant partial locust *LmigOr35* gene sequencing trace and nucleotides sequence

In the migratory locust, the repertoire of the Or gene family (142 genes) has recently been identified [27], and *LmigOr35* is one of 142 olfactory receptor gene family that only has one transcript and is specifically expressed in the antenna of migratory locust. *LmigOr35* contain 5 exons and four introns (Fig. 1a). We designed the targeted site of Cas9 protein at exon 4 near the 3′ splicing site (Fig. 1a). We obtained 22 kinds of mutations of *LmigOr35* using CRISPR/Cas9 system (Additional file 1: Figure S1). The 55 bp nucleotide missing mutation was one of these mutations (Fig. 1b and Additional file 1: Figure S1). The missed 55 bp nucleotides contained 15 bp nucleotides from the intron 3 of *LmigOr35* and 40 bp nucleotides from the exon 4 of *LmigOr35* (Fig. 1b). The 55 bp nucleotide deletion caused the missing of 3′ splice site at the boundary of intron 3 and exon 4 of *LmigOr35* (Fig. 1b). To confirm the wild (+/+) and 55 bp deletion mutants (−/−), we amplified the genome sequences using the primers F1 and R1, and then sequenced the PCR products. We found that the 55 bp deletion homozygous mutant (−/−) had a shorter product compared with the wild types (WT) (+/+) (Fig. 1c). The sequencing of PCR products showed that the 55 bp deletion mutation lost the 55 bp nucleotides at the boundary of intron 3 and exon 4 of *LmigOr35* (Fig. 2). These results indicated that the CRISPR/Cas9 system successfully generated the lacking mutant of 3′ splice site in the migratory locust.

### Missing 3′ splice site at the boundary of intron 3 and exon 4 caused complete exon 4 skipping

**Fig. 3:**
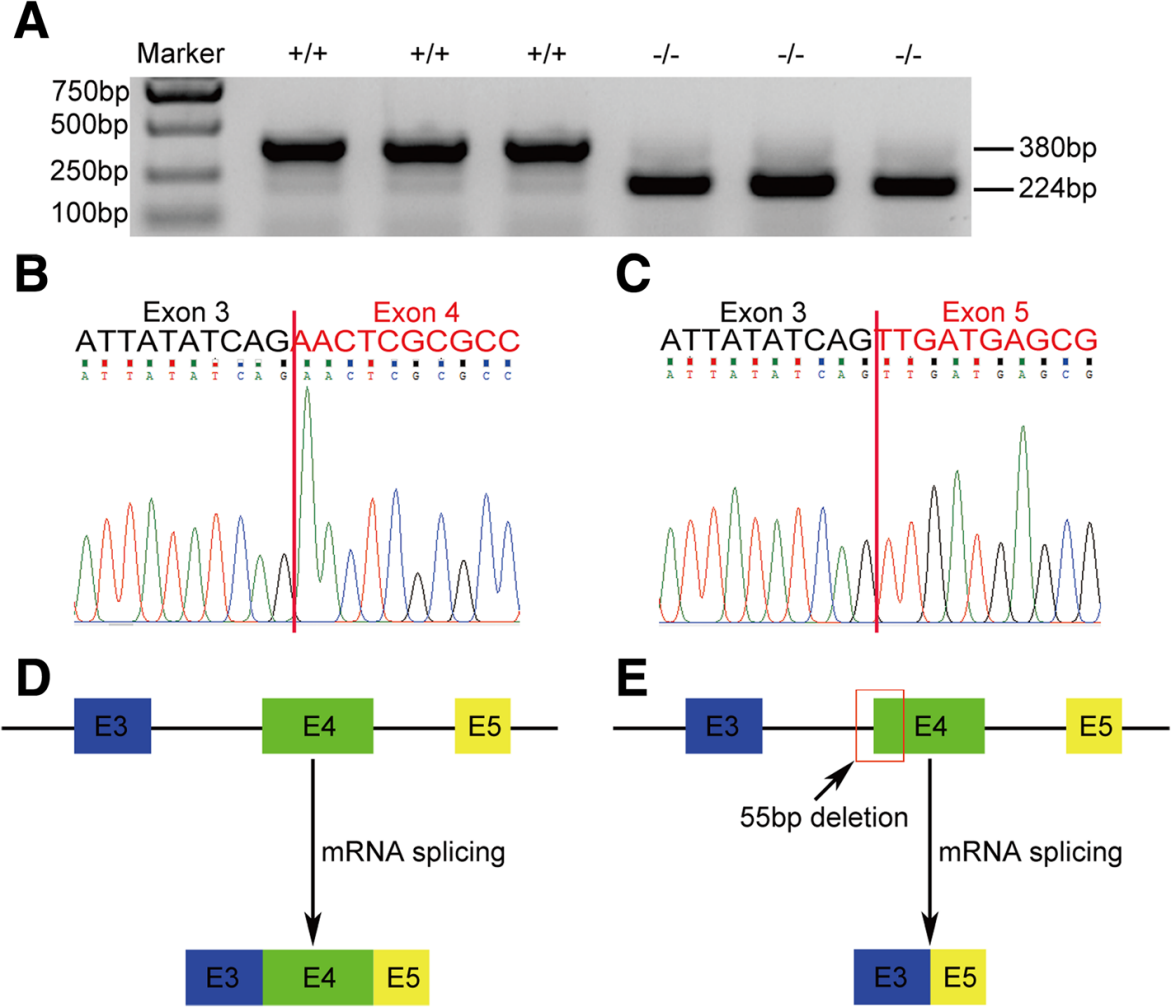
Disruption of 3′ splice site at the boundary of intron 3 and exon 4 of locust LmigOr35 causes complete exon 4 skipping. **a** RT-PCR shows that 156 bp nucleotides are missed in the 55 bp mutant (−/−) locusts compared with the WT (+/+) locusts. **b** WT locusts (+/+) RT-PCR product sequencing shows that exons 3 and 4 of locust LmigOr35 normally combine with each other. **c** 55 bp mutant locusts (−/−) RT-PCR product sequencing shows that exon 3 of locust LmigOr35 combine with exon 5 of locust LmigOr35 and exon 4 is skipped. **d** The WT locust LmigOr35 pre-mRNA splicing. Exons 3, 4, and 5 of locust LmigOr35 normally join with each other after splicing. **e** The 55 bp mutant locust LmigOr35 pre-mRNA splicing. Exons 3 and 5 of locust LmigOr35 combine with each other and exon 4 is skipped after the splicing

We investigated the consequences when the 3′ splice site of *LmigOr35* in the migratory locust was disrupted. We extracted the total RNA from the antenna of WT and mutation lines of the locusts and reversed the total RNA to be cDNA. We amplified exon 3 to exon 5 of *LmigOr35* cDNA. The samples from the WT locusts were a 380 bp band, and the samples from mutant lines only were a 224 bp band. A total of 156 bp losses were observed in the mutant lines and this 156 bp was exactly the sequence of exon 4 (Fig. 3a). A 340 bp band was observed if the mutant samples were normally spliced because 40 bp was missing in exon 4 of the mutant lines. After the sequencing of 224 bp PCR products, we determined that the mutant samples did not contain complete exon 4 compared with the WT samples (Fig. 3b and c). Then, we cloned the PCR products of WT samples and 55 bp mutant determined that all clones in WT samples contained complete exon 4 and no exon 4 was found in all 55 bp mutants (Table 1). Therefore, these results indicated that missing 3′ splice site at the boundary of intron 3 and exon 4 resulted in exon 4 skipping of the mutant samples. In the 3′ splice site deleted mutants, exon 4 was skipped; exons 3 and exon 5 were inappropriately combined.

**Table 1:** The percent of exon skipping in WT, 22 bp mutant and 55 bp mutant locusts

	Analyzed clones (mean/SE)	Exon skipping clones(mean/SE)	Percentage of exon skipping clones(mean/SE)
WT	29/0.58	0/0	0/0
22 bp mutant	27/2.52	7/1.53	26.72/0.07
55 bp mutant	28/1.20	28/1.20	100/0

*SE* standard error

### Deletion of 22 bp nucleotides in exon 4 resulted in stochastic skipping of exon 4

**Fig. 4:**
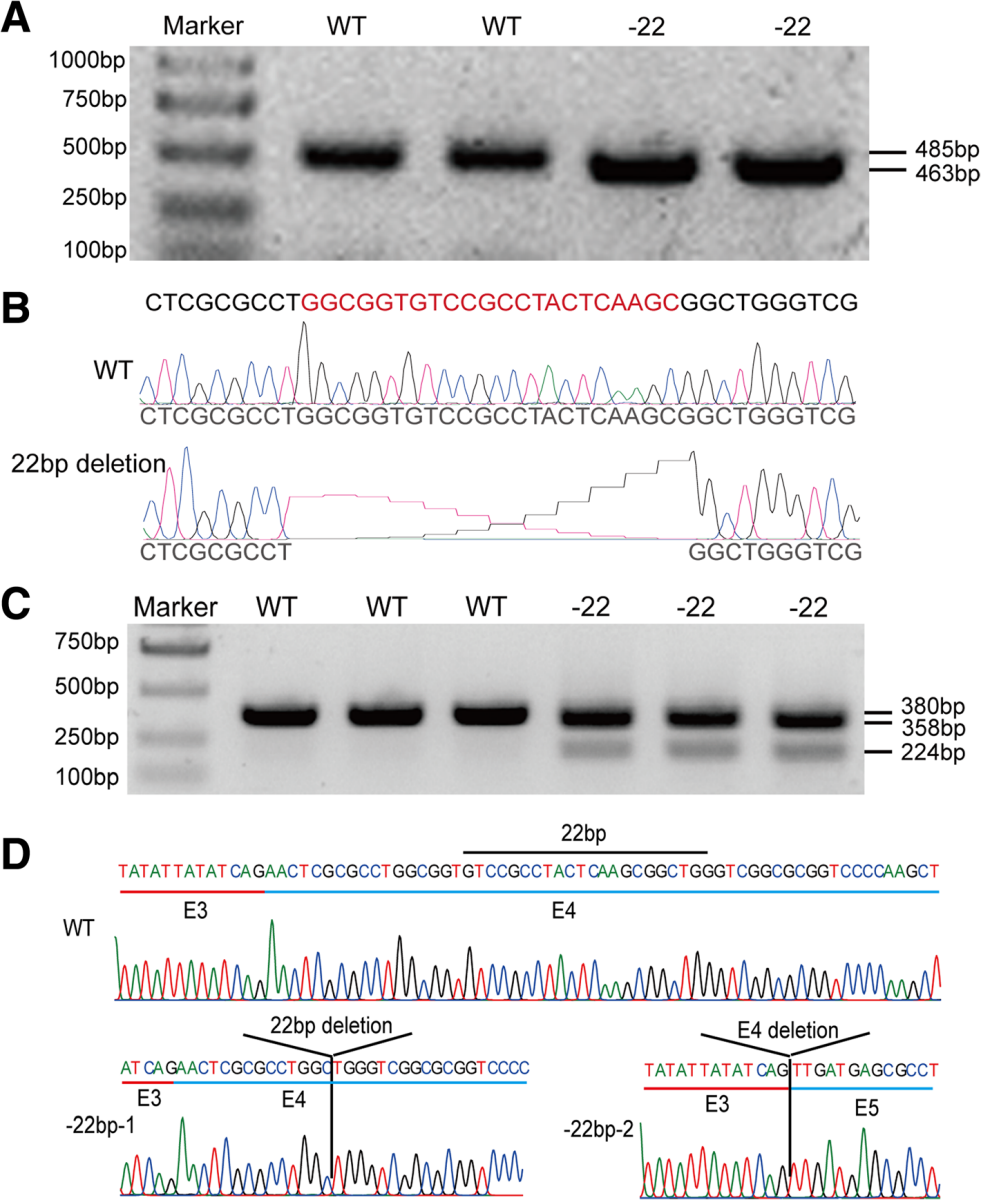
Deleted 22 bp nucleotides in exon 4 of locust LmigOr35 result in alternative splicing and stochastic exon 4 skipping. **a** Genotype of WT and 22 bp mutant (− 22) locusts. The WT locusts obtain a 485 bp brand and the − 22 bp mutants obtain a 463 bp brand. **b** PCR product sequencing of the WT and − 22 bp mutant locust LmigOr35 shows that 22 bp nucleotide is missed in the exon 4 of − 22 bp mutant. **c** RT-PCR shows that WT only has a 380 bp brand and − 22 bp mutants have two brands, namely, the 358 bp and 224 bp brands. **d** RT-PCR product sequencing of the WT and − 22 bp mutant locust LmigOr35. In the WT locust LmigOr35 cDNA, exon 3 normally combines with exon 4. In the − 22 bp mutant locust LmigOr35 cDNA, exon 3 combines with exon 4 (without the − 22 bp nucleotides) in 358 bp brand (− 22 bp − 1), whereas exon 3 combines with exon 5 and exon 4 is skipped in the 224 bp brand (− 22 bp − 2)

We examined whether the lacking mutant 22 bp nucleotides in exon 4 also caused exon 4 skipping. We amplified the targeted sequences using PCR and run the gel. The 22 bp nucleotide deletion mutants had a smaller band (Fig. 4a). The sequencing of small band PCR products showed that the 22 bp nucleotide deletion mutants lacked 22 bp nucleotides in the exon 4 of *LmigOr35* (Fig. 4b). We extracted the total RNA from the antenna of the WT and 22 bp nucleotide deletion mutant of the locusts. The total RNA was reversed into cDNA and amplified exon 2 to exon 5 of *LmigOr35* using primers F2 and R2. We then run the gel and found that there was only one band in the wild type lines, however, there was two bands in 22 bp-nucleotides-deletion mutants (Fig. 4c). After the sequencing of PCR products, we determined that between the two bands in 22 bp nucleotide missing mutants, the first band missed 22 bp nucleotides in exon 4 and the second band missed the entire exon 4 sequences in the 22 bp mutant cDNA products compared with the WT (Fig. 4d). Then, we cloned the PCR products and determined that approximately 26.72% of clones lacked exon 4 (Table 1). These results indicated that the missing 22 bp nucleotides in exon 4 of LmigOr35 resulted in stochastic exon 4 skipping.

## Discussion

Our study shows that genome editing could unintendedly result in exon skipping in the migratory locust. Inducing indels in the exon can lead to stochastic exon skipping, whereas the deletion of the boundary of intron and exon (3′ splice site) can cause the complete exon skipping. These findings prove that complete and stochastic exon skipping can result from the CRISPR-mediated genome editing by deleting different regions of the gene.

Our study showed that the deletion of 22 bp nucleotides resulted in stochastic exon 4 skipping in the exon 4 of *LmigOr35*. Approximately 26% of mRNA displayed the lack of exon 4 in the 22 bp mutant line of the migratory locust. Several recent studies have reported this stochastic exon skipping phenomenon caused by CRISPR-mediated editing of human *FLOT1* and *Ctnnb1* genes in culture cells [13, 15]. In fact, different kinds of point mutations, such as nonsense, missense, and translationally silent mutations, contributed to exon skipping [28–31]. Apart from the splice sites at the intron-exon boundaries, exons also contained splicing elements, such as exon splicing enhancers, bind factors, and exon splicing silencers. Exon skipping caused by indels was because several indel mutations disrupted cis-acting sequences that promoted splicing [16, 28].

**Fig. 5:**
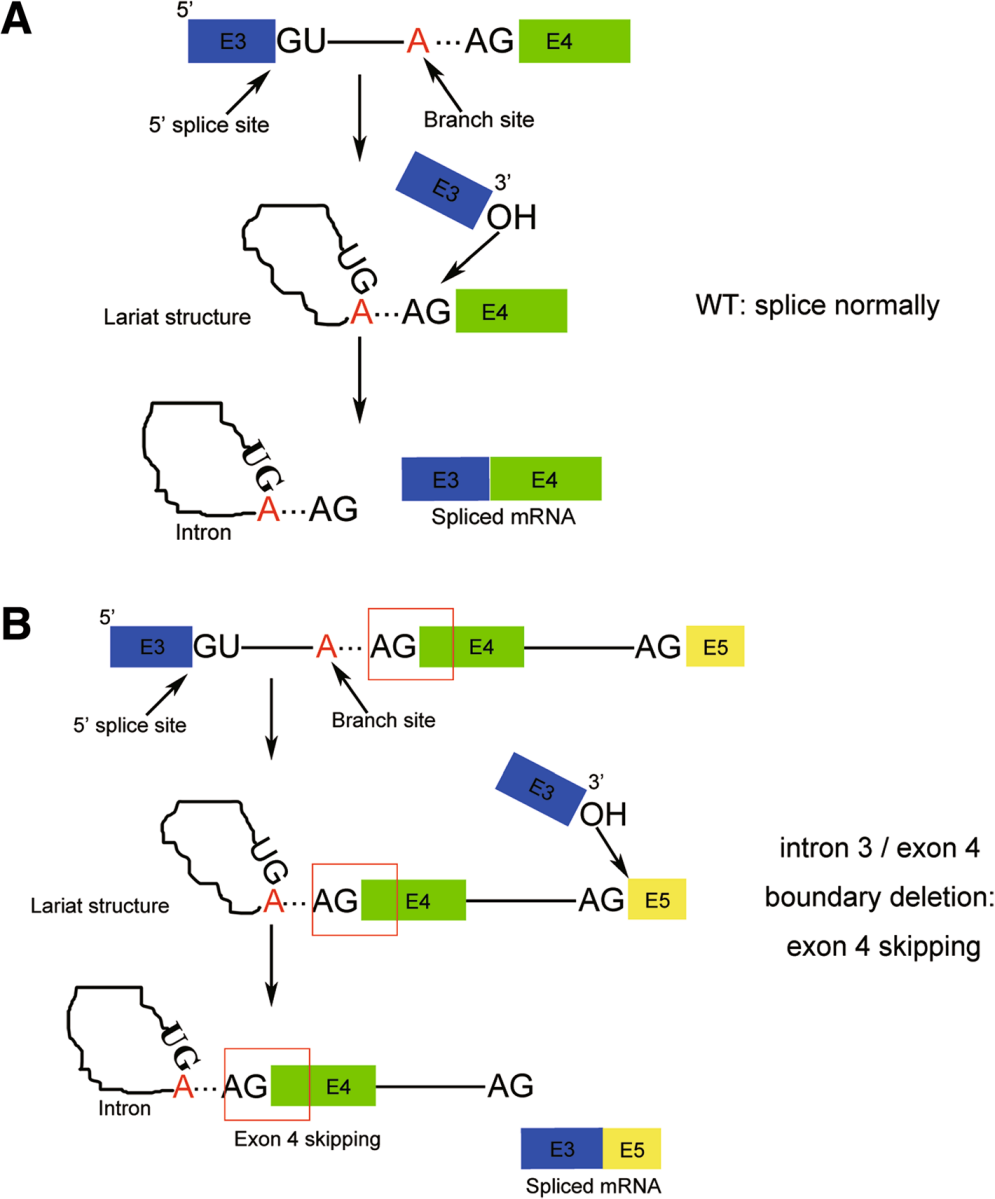
Diagram of the WT and − 55 bp mutant locust LmigOr35 pre-mRNA splicing process. **a** In the WT locust, the branch site attacks the 5′ splice site and the lariat structure is formed. Then, 3’ OH of exon 3 attacks the 3′ splice site by separating intron 3 from exon 4 and combining exon 3 with exon 4. Therefore, intron 3 of LmigOr35 is cut from the pre-mRNA, and exons 3 and 4 combine with each other. **b** In the − 55 bp mutant locust, the lariat structure is normally formed, but the 3′ splice site is disrupted due to the deletion of 55 bp nucleotides. Therefore, 3’ OH of exon 3 attacks the next 3′ splice site located at the boundary of intron 4 and exon 5. Intron 3, exon 4, and intron 4 are cut from the pre-mRNA, and exon 3 combines with exon 5. Exon 4 is skipped during the pre-mRNA splicing. A: the branch site, it locates in the 18–25 bp upstream of the 3′ splice site and not includes in the 55 bp deleted nucleotides

We determined that the boundary deletion of intron 3 and exon 4 (3′ splice site) of *LmigOr35* by CRISPR-mediated editing resulted in complete exon 4 skipping. Other previous studies showed that the frequency in which CRISPR-induced indels caused exon skipping was difficult to predict [13, 15]. Our findings suggested that complete exon skipping was induced by CRISPR-induced indels at the boundary of intron and exon. The indel caused by CRISPR-mediated editing resulted in the deletion of the 3′ splice site located at the boundary of intron 3 and exon 4 of *LmigOr35*. The 3’ OH of exon 3 was unable to recognize the 3′ splice site located at the boundary of intron 3 and exon 4. Therefore, the 3’ OH of exon 3 recognized the next 3′ splice site located at the boundary of intron 4 and exon 5 by attacking the 3′ splice site and separating intron 4 from exon 5. Then, intron 3, exon 4, and intron 4 were released from the pre-mRNA and exon 3 was combined with exon 5 that caused exon 4 skipping (Fig. 5). However, future work should be conducted to prove this hypothesis.

## Conclusions

Our studies revealed the effects of CRISPR-mediated editing on complete or stochastic exon skipping by deleting different regions in vivo. Combined with the results from other studies on cell lines, our observations suggested that CRISPR-mediated editing led to various splicing patterns that depended on the involved splicing regulatory elements. Although exon skipping was the unexpected consequence of CRISPR-mediated editing, it produced mRNA that encoded gain-of-function or partially functional proteins [15]. Currently, the effect of a given indel in exon on pre-mRNA splicing based on genomic sequences was difficult to predict because pre-mRNA splicing regulation was complicated. Thus, we should consider the post-transcript effects when establishing mutant organisms by CRISPR-mediated editing. Moreover, exon skipping caused by CRISPR-mediated editing might be a promising means to investigate the specific exon function by deleting the exon or to treat several genetic diseases caused by splicing mutations [32].

## Methods

### Insects

The locusts used in the experiments were obtained from the breeding stock of *Locusta migratoria* at the Institute of Zoology, CAS, China. All locusts were reared under a 14:10 light/dark photo regime at 30 ± 2 °C and were fed on a diet of fresh greenhouse-grown wheat seedlings and wheat bran.

### Generation of mutant locusts using CRISPR/Cas9 system

The protocol to generate mutant locusts using CRISPR/Cas9 system was previously described [7]. The embryos of locusts were collected from egg pods, washed with 75% ethanol, and were placed on 1% agarose gel. The purified Cas9 protein (Invitrogen, A36496, Massachusetts, USA) and guide RNA were mixed to final concentrations of 400 and 150 ng/μl, respectively (13.8 nL), and were injected in the embryos using a microinjection machine. Then, the embryos were placed in a 30 °C incubator for approximately 14 days until the locusts hatched. The first-instar nymphs were placed in the cages with 14-h-light and sufficient food. We collected part of adult legs and lysed them with a 45 μL NAOH buffer (50 mM) at 95 °C in a PCR machine for 30 min and added 5 μL Tris-HCL (PH = 8.0, 1 M). Then, we used a 2 μL template to amplify the targeted fragments, and we sequenced the fragments to identify whether the mutants were generated. The used primers were designed in introns 3 and 4 of *LmigOr35* as follows: LmigOr35 intron 3-For, GTAAGTTCAGCCTGCTGTAT; LmigOr35 intron 4-Rev, and GTTTCAGCTAGTAGTACGAC. A 485 bp product was obtained from the WT locust after PCR reaction.

### Total RNA extraction

Total RNA was extracted from the antenna of WT and mutant locusts using a TRNzol Reagent (TIANGEN BIOTECH CO., DP405–2, Beijing, China) based on the manuscript description. First, we cut the antennas of one locust, placed the two antennas in a 1.5 mL centrifuge tube, and placed the tube in liquid nitrogen for 20 s. Second, we removed the tube and rapidly ground the antennas with a grinding rod until the antennas achieved a powder form. Third, we added 500 μL of TRNzol Reagent in the tube and thoroughly mixed and we placed the tube at room temperature for 2 min. Fourth, we added 100 μL of chloroform into the tube, thoroughly mixed the solution, and centrifuged it at 12,000 rpm for 10 min at 4 °C. Fifth, 300 μL of supernatant was placed into a new tube, and 300 μL of isopropyl alcohol was added into the tube and thoroughly mixed. Afterward, the tube was placed at − 20 °C for 12 h. Sixth, the tube was centrifuged at 12,000 rpm and 4 °C for 10 min, the supernatant was removed, and 1000 μL of 75% ethanol was added (prepared with nuclease-free water) in the tube followed by thorough mixing. Seventh, the tube was centrifuged at 12,000 rpm and 4 °C for 5 min, the supernatant was removed, and the tube was placed at room temperature for 3 min to dry the RNA precipitation. Eighth, 20 μL of nuclease-free water was added in the tube to dissolve the RNA precipitation and the RNA concentration was measured with Nano-drop 2000.

### RT-PCR and sequencing

RNA was reverse-transcribed with 5X All-In-One RT MasterMix (Applied Biological Materials Inc., G490, British Columbia, Canada) according to the manufacturer’s protocols. First, the RNA templates and 5X All-In-One RT MasterMix were thawed on ice. The solution was gently and thoroughly mixed. Second, the reaction mixture was prepared in a PCR tube on ice (total RNA 2 μg + 5X All-In-One RT MasterMix 4 μL + nuclease-free water to 20 μL). Third, the components were well-mixed and collected by brief centrifugation. Fourth, the tube was incubated in the PCR machine for the reaction (at 25 °C for 10 min, at 42 °C for 50 min for cDNA synthesis, and at 85 °C for 5 min). Fifth, the tube was placed on ice to terminate the reaction. The newly synthesized first-strand cDNA was suitable for immediate downstream applications or for long-term storage at − 20 °C.

For the PCR reaction, we used a 2X TsingKe Master Mix (TsingKe Biotech Co., TSE004, Beijing, China) according to the manufacturer’s protocols. To verify the exon deletion, we use the primers F2 and R2, which were designed in LmigOr35 exon 2 and LmigOr35 exon 5, respectively. The primers sequence are as follows: F2: GTTCTCCTTCAGTTCTTGGG; R2: CATTTGTCATTCACCTGGCG. The WT locust obtained a 380 bp product after PCR reaction. After the PCR reaction, the PCR products executed the gel or sequencing in Beijing TsingKe Biotech Co., Ltd.

## Additional file

Additional file 1: **Figure S1.** Mutant types generated by CRISPR/Cas9 system in exon 4 of locust LmigOr35. WT, wild type; − number, number of nucleotides deletion; Δ number, number of nucleotides substitution; (number), detected number of locusts; green letter, target; yellow letter, PAM; red letter, nucleotides of substitution; gray letter, nucleotides of deletion. (PDF 965 kb)

## Abbreviations

CRISPR/Cas9: Clustered Regularly Interspaced Short Palindromic Repeats/CRISPR associated protein 9
DSB: Double-strand breaks
Indels: Insertion-deletions
NHEJ: Non-homologous end joining
NMD: Nonsense-mediated mRNA decay
Or: Olfactory receptor
PCR: Polymerase chain reaction
