# Supplementary material for: CRISPR/Cas9-mediated genome editing induces exon skipping by complete or stochastic altering splicing in the migratory locust
Source: BMC Biotechnol. 2018 Sep 25;18:60. doi: 10.1186/s12896-018-0465-7 (PMC6156852; doi:10.1186/s12896-018-0465-7)
Supplement: Supplementary file 1 — Figure S1. Mutant types generated by CRISPR/Cas9 system in exon 4 of locust LmigOr35. WT, wild type; − number, number of nucleotides deletion; Δ number, number of nucleotides substitution; (number), detected number of locusts; green letter, target; yellow letter, PAM; red letter, nucleotides of substitution; gray letter, nucleotides of deletion. (PDF 965 kb) [file 12896_2018_465_MOESM1_ESM.pdf]

intron3 exon4

tttcgctttctcgcagAACTCGCGCCTGGCGGTGTCCGCCTACTCAAGCGGCTGGGTCGGCGCGGTCCCCCAGCTGCA	WT	(34)
tttcgctttctcgcagAACTCGCGCCTGGCGGTGTCCGCCTACTCAAGCGGCCGGGTCGGCGCGGTCCCCCAGCTGCA	- 2	(1)
tttcgctttctcgcagAACTCGCGCCTGGCGGTGTCCGCCTACTCAAGCGGCTGGGTCGGCGCGGTCCCCCAGCTGCA	- 16	(1)
tttcgctttctcgcagAACTCGCGACTGGCGGTGTCCGCCTACTCAAGCGC TTGAGTCGGCGCGGTCCCCAAGCTGCA	- 2 Δ1	(1)
tttcgctttctcgcagAACTCGCGCCTGGCGGTGTCCGCCTACTCAAGCGGCTGGGTCGGCGCGGTCCCCCAGCTGCA	- 3	(16)
tttcgctttctcgcagAACTCGCGCCTGGCGGTGTCCGCCTACTCAAGCGGCTGGGTCGGCGCGGTCCCCAAGCTGCA	- 5	(5)
tttcgctttctcgcagAACTCGCGCCTGGCGGTGTCCGCCTACTCAAGCGGCTGGGTCGGCGCGGTCCCCAAGCTGCA	- 4	(3)
tttcgctttctcgcagAACTCGCGCCTGGCGGTGTCCGCCTACTCAAGCGGCTGGGTCGGCGCGGTCCCCAAGCTGCA	- 9	(14)
tttcgctttctcgcagAACTCGCGCCTGGCGGTGTCCGCCTACTCAAGCGGCTGGGTCGGCGCGGTCCCCCAGCTGCA	- 13	(3)
tttcgctttctcgcagAACTCGCGCCTGGCGGTGTCCGCCTACTCAAGCGGCTGGGTCGGCGCGGTCCCCAAGCTGCA	- 11	(1)
tttcgctttctcgcagAACTCGCGCCTGGCGGTGTCCGCCTACTCAAGCGGCTGGGTCGGCGCGGTCCCCAAGCTGCA	- 12	(6)
tttcgctttctcgcagAACTCGCGCCTGGCGGTGTCCGCCTACTCAAGCGGCTGGGTCGGCGCGGCCCCCAAGCTGTA	- 28	(1)
tttcgctttctcgcagAACTCGCGCCTGGCGGTGTCCGCCTACTCAAGCGGCTGGGTCGGCGCGGTCCCCAAGCTGCA	- 5	(5)
tttcgctttctcgcagAACTCGCGCCTGGCGGTGTCCGCCTACGCAAGCGGCTGGGTCGGCGCGGTCCCCAAGCTGCA	- 7	(8)
tttcgctttctcgcagAACTCGCGCCTGGCGGTGTCCGCCTACTCAAGCGGCTGGGTCGGCGCGGTCCCCCAGCTGCA	- 13	(4)
tttcgctttctcgcagAACTCGCGCCTGGCGGTGTCCGCCTACTCAAGCGGCTGGGTCGGCGCGGTCCCCAAGCTGCA	- 6	(6)
tttcgctttctcgcagAACTCGCGCCTGGCGGTGTCCGCCTACTCAAGCGGCTGGGTCGGCGCGGTCCCCAAGCTGCA	- 25	(1)
tttcgctttctcgcagAACTCGCGCCTGGCGGTGTCCGCCTACTCAAGCGGCTGGGTCGGCGCGGTCCCCAAGCTGCA	- 17	(1)
tttcgctttctcgcagAACTCGCGCCTGGCGGTGTCCGCCTACTCAAGCGGCTGGGTCGGCGCGGTCCCCCAGCTGCA	- 23	(1)
tttcgctttctcgcagAACTCGCGCCTGGCGGTGTCCGCCTACTCAAGCGGCTGGGTCGGCGCGGTCCCCAAGCTGCA	- 31	(1)
tttcgctttctcgcagAACTCGCGCCTGGCGGTGTCCGCCTACTCAAGCGGCTGGGTCGGCGCGGTCCCCCAGCTGCA	- 22	(6)
tttcgctttctcgcagAACTCGCGCCTGGCGGTGTCCGCCTACTCAAGCGGCTGGGTCGGCGCGGTCCCCAAGCTGCA	- 38	(1)
tttcgctttctcgcagAACTCGCGCCTGGCGGTGTCCGCCTACTCAAGCGGCTGGGTCGGCGCGGTCCCCAAGCTGCA	- 55	(3)

Figure S1. Mutant types generated by CRISPR/Cas9 system in exon 4 of locust LmigOr35

WT, wild type; - number, number of nucleotides deletion; Δ number, number of nucleotides substitution; (number), detected number of locusts; green letter, target; yellow letter, PAM; red letter, nucleotides of substitution; gray letter, nucleotides of deletion.
